# Complete mitochondrial genome and phylogenetic analysis of *Chloris chloris* (Passeriformes: Fringillidae)

**DOI:** 10.1080/23802359.2024.2410468

**Published:** 2024-10-01

**Authors:** Xiaodong Gao, Jincheng Liu, Xinyue Zhang, Tong Zhao

**Affiliations:** a College of Life Science, Qufu Normal University, Qufu, PR China; b Department of Student Affairs, Qufu Normal University, Qufu, PR China

**Keywords:** Chloris chloris, mitochondrial genome, high-through metagenomic data

## Abstract

In this study, we employed high-throughput metagenomic data to assemble the mitochondrial genome (mitogenome) of the European greenfinch (*Chloris chloris*; Linnaeus 1758). The circular mitogenome was 16,813 base pairs (bp) in length, containing 13 protein-coding genes (PCGs), 22 tRNA genes, 2 rRNA genes, and 1 control region. The base composition of the mitogenome is 30.6% A, 30.7% C, 14.2% G, and 24.5% T, resulting in a GC content of 44.9%. Phylogenetic analysis, utilizing the concatenation of the 13 mitochondrial PCGs from 32 related species of the order Passeriformes, indicated a closer relationship between *C. chloris* and *C. sinica*. Moreover, the genus *Chloris* was closely related to the genera of *Serinus*, *Crithagra*, *Carduelis*, and *Acanthis*. This mitogenomic data of *C. chloris* not only be helpful for species identification but also facilitates our understanding of the evolutionary relationship among different species in genus *Chloris*, which experienced rapid radiation evolution.

## Introduction

**Figure 1.**
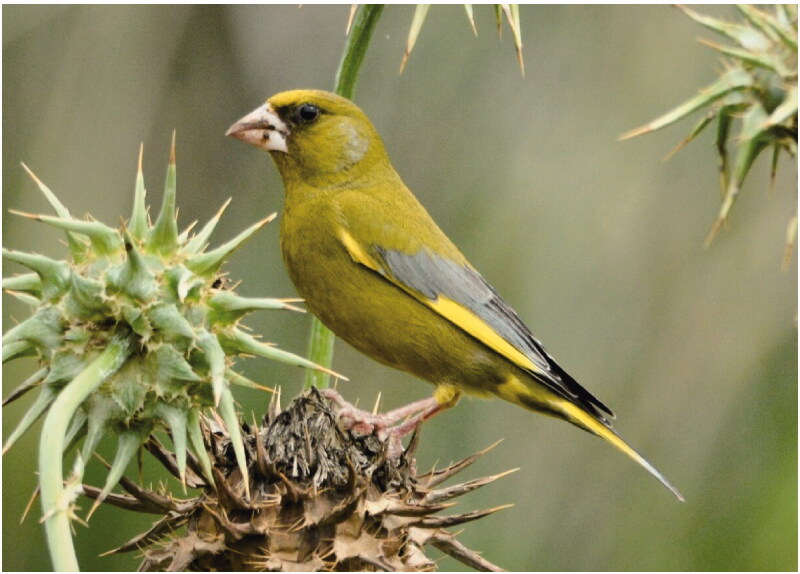
Reference image of the *Chloris chloris* (photographer: Karim Haddad, email: karim241267@yahoo.fr, used with permission). This photograph was captured in May 2023. The original photograph is accessible to the public on iNaturalist (https://www.inaturalist.org/observations/222699247).

The European greenfinch, scientifically known as *Chloris chloris* (Figure 1), is a small passerine bird in the Fringillidae family. *C. chloris* inhabits an extremely large range across Eurasian, and has been introduced to southeastern South America, Australia, and New Zealand (BirdLife International 2018). The European greenfinch is characterized by its compact build and distinctive cone-shaped beak. It exhibits adaptability to various habitats, such as suburban gardens, farmlands, woodlands, plantations, and orchards. Although predominantly herbivorous, the European greenfinch may supplement its diet with insects during the warmer seasons (Hanmer et al. 2022). Currently, the greenfinch holds the status of ‘Least Concern’ (LC) on the IUCN Red List, with its population size and trend being evaluated as ‘Stable’ (BirdLife International 2018). However, due to human activities and pathogen infections, the greenfinch population has declined steadily, e.g. in the UK, an estimated reduction of up to 66% of the breeding population and a continuous decline is still going on (Lawson et al. 2018). Despite the fact that the genus *Chloris* is one of the most speciose genus of the family Fringillidae, complete mitochondrial genome from only one species was determined to date namely, *C. sinica* (Linnaeus 1766), which is mainly distributed in Eastern Asia (Josep et al. 2010). Herein, we report and describe the complete mitogenome of *C. chloris* for the first time and conduct a comprehensive phylogenetic analysis to describe the position of *C. chloris* and genus *Chloris* in family Fringillidae.

## Materials and methods

The mitogenome of *C. chloris* was reconstructed utilizing publicly available high-throughput metagenomic data and this strategy has been proven to be efficient for other species (Hervé and Brune 2017; Wu et al. 2021). An individual fecal specimen from *C. chloris* used in this study was collected in Hungary (geographic coordinates: latitude 47.5 and longitude 19.1) (Youngblut et al. 2020). This sample was deposited at the Max Planck Institute for Developmental Biology (https://www.bio.mpg.de/2923/en, Nicholas D. Youngblut, nyoungblut@tuebingen.mpg.de) under the voucher number X172_European_Greenfinch. Metagenomic data of this sample (NCBI SRA accession number: ERR4083919) was obtained as previously described (Youngblut et al. 2020). Briefly, DNA was extracted from the fecal sample using the PowerSoil DNA isolation kit (MoBio Laboratories, USA). Subsequently, metagenomic libraries were prepared and sequenced on an Illumina HiSeq3000 platform employing the 2 × 150 paired-end sequencing mode. The quality-trimmed sequencing reads from the metagenomic data were collected, and mitochondrial reads were extracted and assembled using MitoZ v3.6 (Meng et al. 2019). After a manual inspection, the resulting mitogenome was annotated and visualized using OGDRAW v1.3.1 (Greiner et al. 2019). The annotated mitogenome was deposited in GenBank under the accession number of OR220880.

To examine the phylogenetic relationship between *C. chloris* and its relatives, we conducted a thorough search of the NCBI GenBank database and obtained the mitogenomes of 30 species belonging to the family Fringillidae, with each species representing a distinct genus. When comparing the mitogenome of *C. chloris* with the other 30 mitogenomes from the Fringillidae family using GenBank BLAST searches, we ensured a minimum query coverage of 98% and a percent identity of 84.8%. Phylogenetic analysis using maximum likelihood (ML) of *C. chloris* and its relatives was performed using IQ-TREE2 (Nguyen et al. 2015) based on the sequences of 13 mitochondrial PCGs. Sequence of each PCG was separately aligned using MUSCLE v3.8.31 (Edgar 2004). The best-fit substitution model for each alignment was determined using ModelFinder model (Kalyaanamoorthy et al. 2017). Branch reliability for the ML tree was assessed using UFBoot2 with 1,000 bootstrap replicates (Hoang et al. 2018). Phylogenetic tree was also reconstructed using the Bayesian inference (BI) approach with MrBayes v3.2.7 (Ronquist and Huelsenbeck 2003). BI analysis was performed as follow: Markov chains were run for 1,000,000 generations with trees being sampled every 500 generations, four chains and a burn-in step for the first 500 generations.

## Results

**Figure 2.**
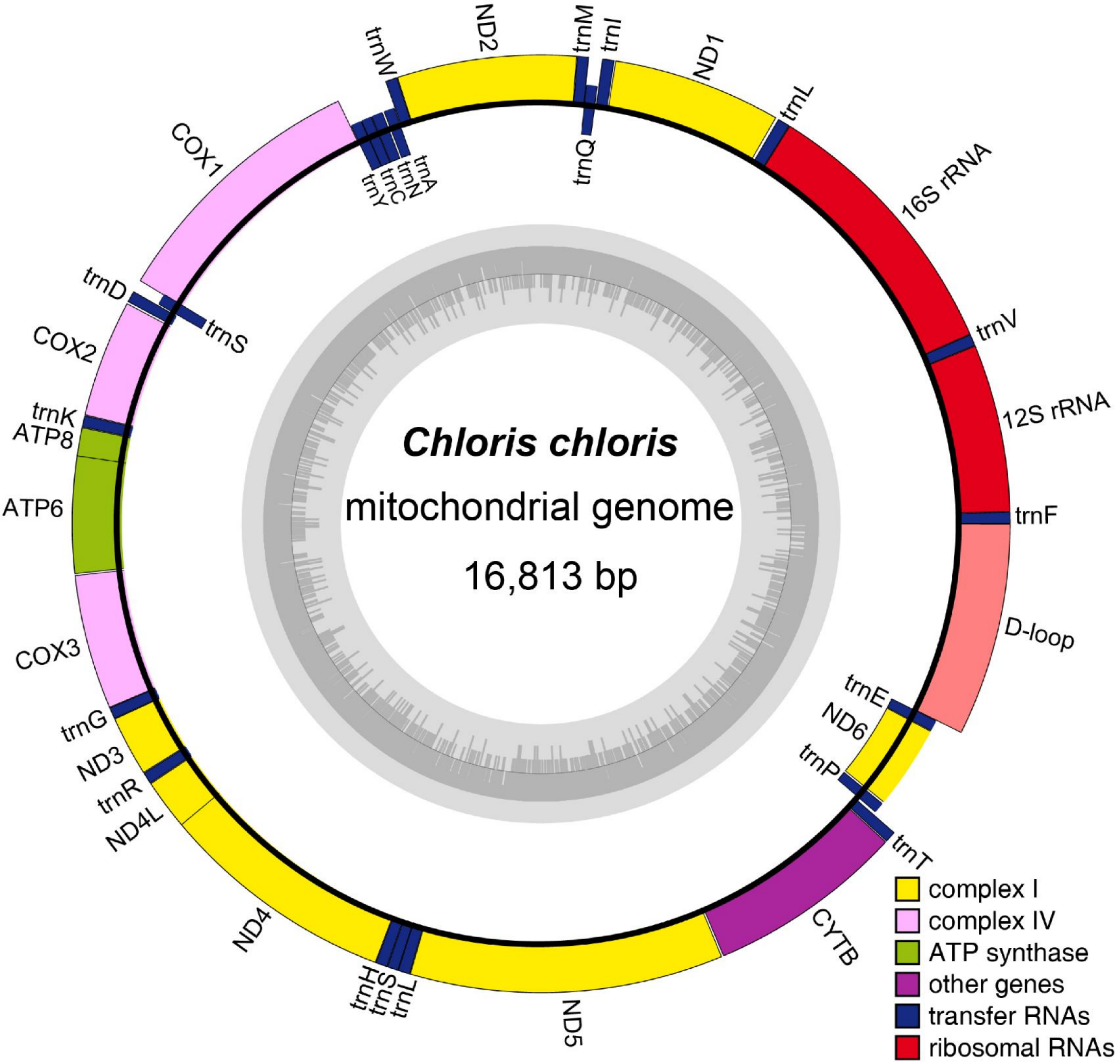
The mitochondrial genome map of *Chloris chloris*, drawn by OGDRAW v1.3.1 (Greiner et al. 2019).

The complete mitogenome of *C. chloris* spans 16,813 bp and contains 13 PCGs, 2 rRNA genes, 22 tRNA genes, a light-strand replication origin (O_L_) and a putative long noncoding region called the control region (D-loop) (Figure 2). This genetic arrangement adheres to the typical structure of vertebrate mitochondrial genes. The overall base composition is 30.6% for A (5151 bp), 14.2% for G (2386 bp), 30.7% for C (5158 bp) and 24.5% for T (4118 bp), with a slight majority of AT content (55.1%; 9,269 bp). Eleven PCGs began with an ATG start codon while just two, for *COX1* started with GTG and *ND3* started with ATA. Among the 13 PCGs, *ND5* is the longest (1819 bp), while *ATP8* is the shortest (169 bp). The two rRNA genes (*12S rRNA* and *16S rRNA*) are 977 bp and 1,599 bp in length respectively, located between *tRNA^Phe^* and *tRNA^Leu^*, and separated by the *tRNA^Val^* gene. Most of the mitochondrial genes were encoded on the H-strand, with the exception of one PCG (*ND6*) and eight tRNA genes (*tRNA^Gln^*, *tRNA^Ala^*, *tRNA^Asn^*, *tRNA^Cys^*, *tRNA^Tyr^*, *tRNA^Ser^*, *tRNA^Pro^*, and *tRNA^Glu^*) that were encoded on the L-strand. The control region (D-loop) was 1237 bp in length, located between the tRNA^Glu^ and tRNA^Phe^ genes.

## Discussion

**Figure 3.**
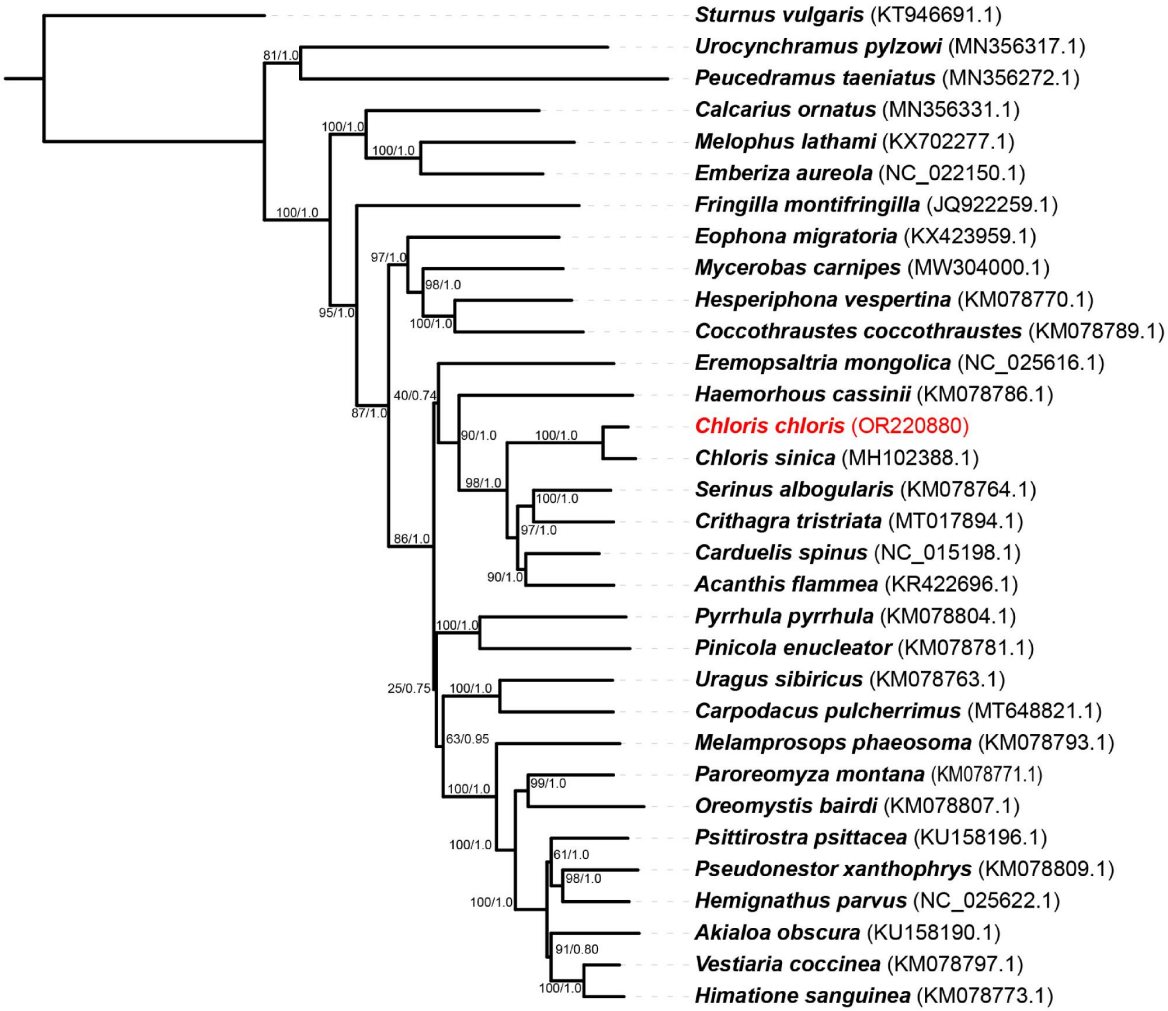
Phylogenetic tree of selected species of family Fringillidae based on maximum likelihood (ML) using 13 protein-coding genes. Similar phylogeny was obtained when Bayesian inference (BI) method was employed. Bootstrap values and posterior probabilities were shown on the branches. The number after each species name is the corresponding GenBank accession number. The following sequences were used to infer the tree: *Acanthis flammea* (KR422696.1), *Akialoa obscura* (KU158190.1), *Calcarius ornatus* (MN356331.1), *Carduelis spinus* (NC_015198.1), *Carpodacus pulcherrimus* (MT648821.1), *Chloris sinica* (MH102388.1), *Coccothraustes coccothraustes* (KM078789.1), *Crithagra tristriata* (MT017894.1), *Emberiza aureola* (NC_022150.1), *Eophona migratoria* (KX423959.1), *Eremopsaltria mongolica* (NC_025616.1), *Fringilla montifringilla* (JQ922259.1), *Haemorhous cassinii* (KM078786.1), *Hemignathus parvus* (NC_025622.1), *Hesperiphona vespertina* (KM078770.1), *Himatione sanguinea* (KM078773.1), *Melamprosops phaeosoma* (KM078793.1), *Melophus lathami* (KX702277.1), *Mycerobas carnipes* (MW304000.1), *Oreomystis bairdi* (KM078807.1), *Sturnus vulgaris* (KT946691.1), *Paroreomyza Montana* (KM078771.1), *Peucedramus taeniatus* (MN356272.1), *Pinicola enucleator* (KM078781.1), *Pseudonestor xanthophrys* (KM078809.1), *Psittirostra psittacea* (KU158196.1), *Pyrrhula pyrrhula* (KM078804.1), *Serinus albogularis* (KM078764.1), *Uragus sibiricus* (KM078763.1), *Urocynchramus pylzowi* (MN356317.1), *Vestiaria coccinea* (KM078797.1) and *Chloris chloris* (OR220880).

Despite their great diversity, the internal phylogeny of family Fringillidae has been debated (Zuccon et al. 2012). Phylogenetic analysis of *C. chloris* and its relatives was performed using ML and BI methods based on the sequences of 13 mitochondrial PCGs from 31 species of the family Fringillidae and one species of the family Sturnidae, which was used as the outgroup (Figure 3). Our sampling included almost all the mitogenomes at the genus level of family Fringillidae, which were available in GenBank. Individual gene alignments were then concatenated into a partitioned supermatrix encompassing 11,361 bp. Phylogenetic tree demonstrated that *C. chloris* is most closely related to *C. sinica*. Moreover, the genus *Chloris* was closely related to the genera of *Serinus*, *Crithagra*, *Carduelis*, and *Acanthis* (Figure 3). Our study provides a valuable genetic resource for future investigations into species identification and phylogenetic position of this species commonly found across Europe.

## Supplementary Material

Supplemental Material

## Data Availability

The metagenome sequencing data used for assembling the mitochondrial genome of *C. chloris* are openly available in SRA of NCBI (https://www.ncbi.nlm.nih.gov/) under the accession number ERR4083919. The associated BioProject and Bio-Sample numbers are PRJEB38078 and SAMEA6809406 respectively. The genome sequence data that support the findings of this study are openly available in GenBank of NCBI under the accession no. OR220880.
