# Supplementary figures and images for: Complete mitochondrial genome and phylogenetic analysis of Chloris chloris (Passeriformes: Fringillidae)
Source: Mitochondrial DNA B Resour. 2024 Oct 1;9(10):1327–30. doi: 10.1080/23802359.2024.2410468 (PMC11445922; doi:10.1080/23802359.2024.2410468)

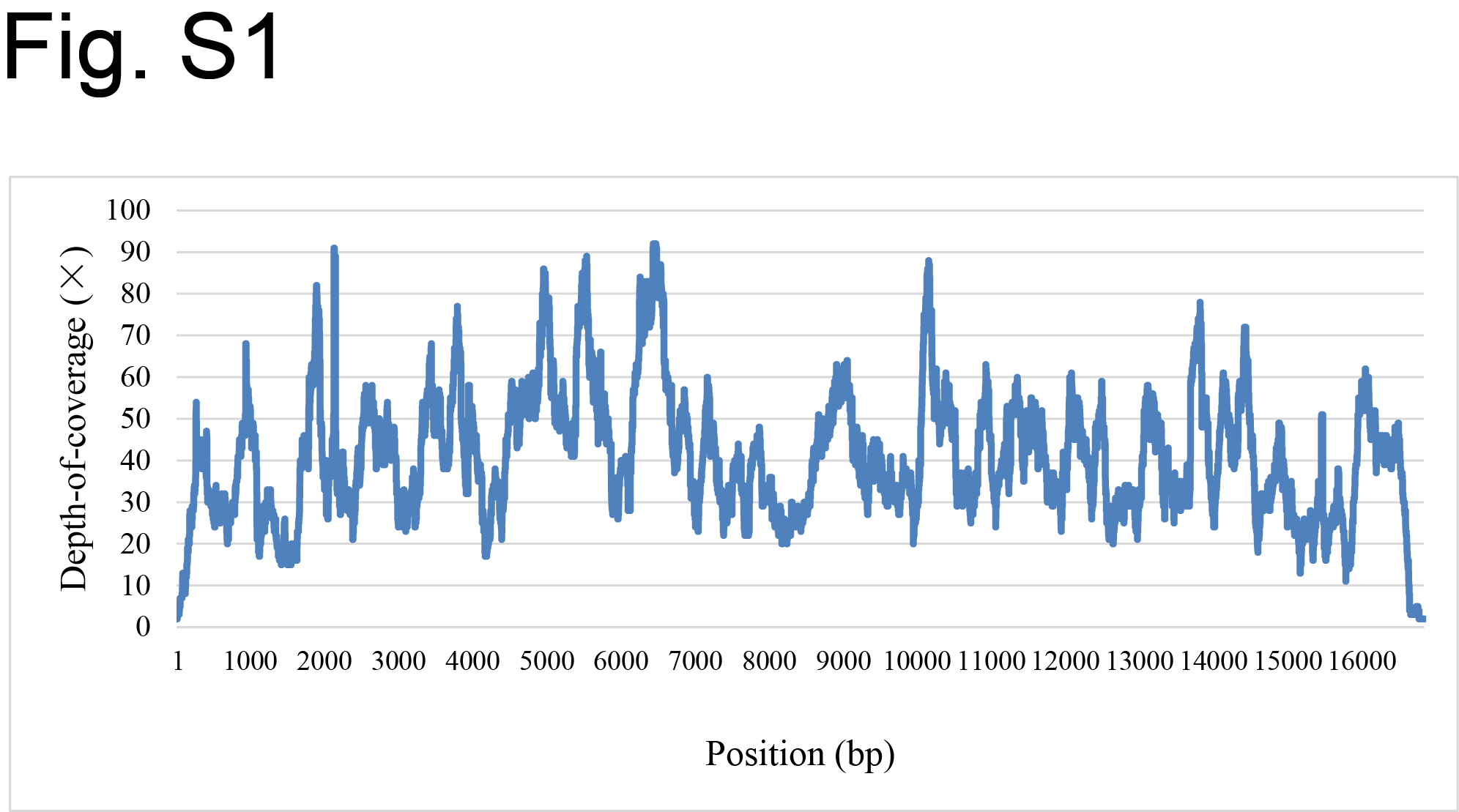

Supplement: Supplemental Material [file TMDN_A_2410468_SM3118.jpg]
